# Valorisation of Pea Pod by‐Product Through Lactic Acid Bacteria Fermentation and Application in Cookie Production

**DOI:** 10.1002/fsn3.72245

**Published:** 2026-08-13

**Authors:** Dilara Devecioglu, Berfin Kaya, Neris Danç, Aysegul Mutlu‐Ingok, Funda Karbancioglu‐Guler

**Affiliations:** ^1^ Faculty of Chemical and Metallurgical Engineering, Department of Food Engineering Istanbul Technical University Maslak Istanbul Türkiye; ^2^ Department of Food Processing, Akçakoca Vocational School Düzce University Akçakoca Düzce Türkiye

**Keywords:** by‐product, cookie, fermentation, flour, pea pod

## Abstract

Peas are widely used in various food processes, particularly in canned food production. However, large quantities of pea pods are generated as by‐products, despite their considerable nutritional potential. This study contributes to the existing literature by comprehensively assessing pea pods as a value‐added by‐product. Pea pod flour (PPF) was fermented using *Lactiplantibacillus plantarum* DD_T_B61 and *Lacticaseibacillus casei* DD_B_B71. Based on its high total phenolic content (11.31 ± 0.36 mg GAE/g) and strong antioxidant activity, PPF fermented by *Lacticaseibacillus casei* DD_B_B71 (F‐PPF) for 24 h was selected for cookie production. Cookies formulated with unfermented (PPF‐C) and fermented flours (F‐PPF‐C) were evaluated for physicochemical, textural, and bioactive properties. Although TPC values in PPF‐C and F‐PPF‐C were lower than expected, both cookie formulations exhibited significantly higher antioxidant activity than wheat flour cookies. The incorporation of fermented PPF increased tartaric, acetic, and oxalic acid contents, while lactic and citric acids were detected only in F‐PPF‐C. Sensory analysis indicated that PPF‐C was preferred for odor, texture, and overall acceptability, whereas F‐PPF‐C performed better in terms of color and appearance. These differences suggest that sensory acceptance could be further improved through formulation optimisation. Overall, the findings highlight the potential of F‐PPF to enhance the nutritional value of bakery products while contributing to by‐product valorisation.

## Graphical abstract

Pea pod by‐products were valorised through fermentation with lactic acid bacteria and applied in cookie production. Fermentation enhanced antioxidant activity, particularly after 24 h with *L. casei* DD_B_B71. Cookies with fermented flour showed improved bioactive profiles, though sensory optimisation is needed, highlighting a promising approach for sustainable, functional bakery product development.

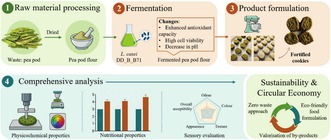

## 1 Introduction

Pea ( *Pisum sativum* ), a member of the pulse family, plays a significant role in global production (Bento et al. 2025). In 2024, green pea production was dominated by mainland China, followed by India, Pakistan, and France (FAOSTAT 2026). Pea pods (PP) constitute a significant portion of the total weight (approximately 35%–40%) (Nasir et al. 2024). During pea processing, large quantities of PP are generated as by‐products, and their disposal results in substantial economic losses (Di Biase et al. 2025). In addition, challenges in waste management in the food industry highlight the importance of utilizing such by‐products as value‐added ingredients. Their functional potential has driven growing research interest, making PP valorisation an emerging trend in food science (Mateos‐Aparicio et al. 2010).

PP contains notable amounts of protein, sugars, and minerals, and is recognized as an important source of dietary fiber (Nasir et al. 2024). Their dietary fiber content has been reported as 58.6 g/100 g DM, while the protein, fat, and ash contents are 10.8, 1.3, and 6.6 g/100 g DM, respectively (Mateos‐Aparicio et al. 2010). Due to this high fiber content, PP powder has been suggested for use in dietetic food products (Nasir et al. 2023). Additionally, appreciable levels of potassium and iron have been reported (Mateos‐Aparicio et al. 2010) along with total sugars (4.8%) and phenolic compounds (0.3% DM) (Wadhwa et al. 2006). Considering their cost‐effectiveness, significant contribution to the total weight, and notable phenolic content and antioxidant activity, PP has the potential to be utilized as a valuable by‐product (Babbar et al. 2014).

Moreover, the comparatively low levels of anti‐nutritional factors reported in PPF represent a key advantage, supporting its utilization as a low‐cost ingredient in food systems (Ben Said et al. 2025). As the moisture content was relatively higher in PP (73.5%) (Nimbalkar et al. 2018), its conversion into powder form is advantageous to reduce microbial spoilage and extend shelf life. It has been suggested for use in the bakery industry due to its dietary and structural benefits (Nasir et al. 2024). In the literature, pea by‐products such as peels and pods have been evaluated for a wide variety of properties in different forms, including powder, extract, and flour (Pooja et al. 2022; Babbar et al. 2014; Ben Said et al. 2025; Castaldo et al. 2022; Estivi et al. 2025; Hadrich et al. 2014; Kaur et al. 2023; Mateos‐Aparicio et al. 2010, 2012). It is also possible to utilize pea by‐products in muffins (Pooja et al. 2022), bread (Estivi et al. 2025; González‐Montemayor et al. 2021), flatbread (Sik et al. 2024), cookies (Kaur et al. 2023; Nasir et al. 2023), and instant soup formulations (Hanan et al. 2020), as well as for the production of polysaccharides (Belghith‐Fendri et al. 2018) and xylooligosaccharides (Cebin et al. 2021).

Some processing methods, particularly fermentation, have been shown to enhance techno‐functional properties and improve the nutritional value of pulses (Bento et al. 2025) while promoting the valorisation of their by‐products (Nartea et al. 2023). Although fermentation has been widely investigated in legumes and their by‐products, research focusing on PP remains limited despite their potential for biological and functional enhancement through fermentation. In particular, the partial substitution of wheat flour (WF) with legume flours has received considerable attention, whereas the potential use of legume by‐products in bakery products remains underexplored. Therefore, this study provides a preliminary assessment of the use of fermented pea pod flour for cookie production by investigating its fermentation using two different lactic acid bacteria (LAB) isolates.

## 2 Material and Methods

### 2.1 Samples and Microorganisms

PP purchased from the local market in Türkiye were freeze‐dried, ground, and sieved using a 224 μm sieve (Retsch, Germany) to remove particles of undesirable size and then stored at −40°C. Two different LAB (*Lactiplantibacillus plantarum* DD_T_B61 and *Lacticaseibacillus casei* DD_B_B71) isolated from the local sources were selected for fermentation (Devecioglu et al. 2026). The stock cultures were maintained in 20% (w/v) glycerol solution at −80°C.

### 2.2 Flour Fermentation

The fermentation of PPF by two different isolates was conducted according to the method of Pei et al. (2022) with minor modifications. Briefly, PPF suspension (1:5 w flour/v water) was sterilized at 115°C for 15 min. After cooling to room temperature, LAB (1 × 10^8^ CFU/mL, 3%) were inoculated into the suspension. Fermentation was carried out at 37°C with agitation at 100 rpm, and samples were collected at 0, 6, 24, and 48 h. A non‐inoculated suspension was used as the control. Microbial viability and pH were monitored throughout the fermentation period. All fermented and control samples were freeze‐dried and stored at −20°C until further analysis. Fermentation of the flour samples was performed in three independent experimental replicates, and each experimental replicate was analyzed at least in duplicate.

### 2.3 Characterization of Flours

#### 2.3.1 Extract Preparation

Collected and dried samples during fermentation and produced cookies were extracted according to Capanoglu et al. (2008) with slight modification. Samples (50–200 mg) were mixed with 2 mL of 80% v/v methanol solution. After 15 min sonication in an ultrasonic water bath (Bandelin Sonorex), the supernatant was collected by centrifugation (2500 rpm, 10 min). The extraction procedure was repeated for the remaining pellet, and all collected liquid parts were combined and stored for further analysis.

#### 2.3.2 Antioxidant Activity

The antioxidant capacity of the samples was determined using the 2,2‐diphenyl‐1‐picrylhydrazyl (DPPH) and cupric ion reducing antioxidant capacity (CUPRAC) assays with minor modifications (Kumaran and Joel Karunakaran 2006). For the DPPH assay, 2 mL of 0.1 mM DPPH solution prepared in 100% methanol was mixed with 100 μL of the sample extract. The mixture was incubated in the dark at room temperature for 30 min. The absorbance was measured at 517 nm using a UV–Vis spectrophotometer (BioTek Instruments, USA). For the CUPRAC assay, 1 mL of 10 mM CuCl_2_, 1 mL of 7.5 mM neocuproin, 1 mL of 1 M NH_4_Ac (pH 7.0), and 1 mL of distilled water were mixed with 100 μL of the sample extract. After incubation at room temperature in the dark for 30 min, the absorbance was recorded at 450 nm using a UV–Vis spectrophotometer. The results of both assays were expressed as mg Trolox equivalents (TE)/g of sample extract.

#### 2.3.3 Total Phenolic Content

The total phenolic content was determined using the Folin–Ciocalteu method, with minor modifications as described by Singleton and Rossi (1965). Briefly, 100 μL of the sample extract was mixed with 750 μL of 10% (v/v) Folin–Ciocalteu reagent and 600 μL of 7.5% (w/v) sodium carbonate solution. The reaction mixture was incubated at room temperature in the dark for 90 min. The absorbance was measured at 765 nm using a UV–Vis spectrophotometer. The results were expressed as mg gallic acid equivalents (GAE)/g of sample extract.

#### 2.3.4 Color Analysis

Color changes were calculated by L*, a* and b* values of PPF and F‐PPF in comparison with L*, a* and b* values of WF (Kaur et al. 2023).

$$\mathrm{\Delta E}=\sqrt{{\left(L_{1}-L_{2}\right)}^{2}+{\left(a_{1}-a_{2}\right)}^{2}+{\left(b_{1}-b_{2}\right)}^{2}}$$

where L_1_, a_1_, b_1_ value of WF, L_2_, a_2_, b_2_ value of PPF and F‐PPF.

#### 2.3.5 Organic Acid Composition

The organic acid composition of samples was determined by the modified methods of Devecioglu et al. (2024) and Gao et al. (2022). Sample preparation was carried out according to the method described by Chiş et al. (2020) with slight modifications. In brief, 1 g of the sample was homogenized with 5 mL of distilled water, then vortex‐mixed for 1 min. The mixture was subjected to ultrasonic extraction at 50°C for 2 h. After extraction, the samples were centrifuged at 9000 rpm for 10 min, and the resulting supernatants were collected and filtered before HPLC analysis. The Agilent Technologies 1100 HPLC system, composed of a degasser (G1379A), a quaternary pump (G1311A), an autosampler (G1313A ALS), a column heater (G1316A TCC), and a diode‐array detector (G1315D), was used. The HPLC system was equipped with a C18 column (Supelco, USA, 250 mm × 4.6 mm, 5 μm) maintained at 30°C. The mobile phase consisted of HPLC‐grade water containing 0.1% phosphoric acid and methanol (97.5:2.5, v/v) under isocratic conditions. The flow rate was set at 0.5 mL/min, and detection was performed at 210 nm.

### 2.4 Cookie Production

Three different cookie formulations were prepared using 100% wheat flour (control, WF‐C), 100% unfermented pea pod flour (PPF‐C), or 100% pea pod flour fermented by *Lacticaseibacillus casei* DD_B_B71 (F‐PPF‐C). Briefly, 75 g of butter (at room temperature) and 35 g of sugar were mixed until a homogeneous mixture was obtained. Then, 100 g of flour was incorporated and mixed until a uniform dough was formed. The cookie dough was divided into small portions (5–7 g each) and molded. The cookie pieces were baked at 170°C for 10–15 min. After baking, the cookies were allowed to cool to room temperature before further analysis. All cookies were produced in a single batch to minimize inter‐batch variability, and all the experiments were performed in triplicate.

### 2.5 Cookie Analysis

The antioxidant activity, total phenolic content, color parameters, and organic acid content of the cookies were determined using the same analytical methods described for the flour samples.

Water activity (a_w_) was determined by placing crushed cookie samples into the sample pan and measuring them at 25°C. Moisture content was determined according to the AOAC (2000) method. Briefly, 1–2 g of each sample was weighed into pre‐dried and pre‐weighed containers and dried in an oven at 105°C for 3–4 h. After drying, the samples were cooled to room temperature and reweighed. Moisture content was calculated using the following equation:

$$\text{Moisture}\;\left(\%\right)=\frac{W_{1}-W_{2}}{W_{1}}\times 100$$

where W_1_ and W_2_ are the weights of the samples before and after drying, respectively.

The diameter (D) and thickness (T) of the cookies were measured at three different points using a digital micrometer (Aurora Tools, 0–25 mm), and the spread ratio (D/T) was subsequently calculated.

Cookie weight was recorded before and after baking, and baking loss (%) was calculated based on the reduction in weight after baking.

Cookie hardness was determined using a texture analyzer (Brookfield CT3) equipped with a TA40 probe. The test conditions were as follows: a 4500 g load cell capacity, a pre‐test speed of 2 mm/s, a test speed of 1 mm/s, and a target distance of 12 mm. Hardness was defined as the maximum force (g) required to break the samples. The results were expressed as the mean of three independent measurements.

Sensory evaluation was conducted to assess the quality attributes of three cookie formulations: WF‐C, PPF‐C, and F‐PPF‐C. The samples were coded with random three‐digit numbers and presented to the panelists in a randomized order. Sensory attributes, including color, appearance, texture, odor, and overall acceptability, were evaluated using a nine‐point hedonic scale (1 = dislike extremely; 9 = like extremely) by 15 semi‐trained panelists aged 25–45 years (Castro Delgado et al. 2020; Sivaraman et al. 2023). Before sensory evaluation, all participants provided voluntary informed consent. Participation was voluntary and anonymous, and all procedures were conducted in accordance with the principles of confidentiality and applicable institutional guidelines. Formal ethics committee approval was not required.

### 2.6 Statistical Analysis

All statistical analyses were performed using SPSS Version 24.0 (IBM Corp., Chicago, IL, USA). The results were expressed as mean ± standard deviation. Prior to one‐way analysis of variance (ANOVA), the assumptions of normality and homogeneity of variances were assessed using the Shapiro–Wilk and Levene's tests, respectively. Then, data were analyzed by ANOVA, followed by Tukey's b multiple comparison test. Differences were considered statistically significant at *p* < 0.05. Pearson correlation analysis was used to evaluate the linear relationships among the tested variables of PPF‐C and F‐PPF‐C. The correlation coefficient (*r*) and corresponding *p*‐values were calculated to assess the strength and significance of the associations. Significance levels were set at *p* < 0.05 and *p* < 0.01.

## 3 Results and Discussion

### 3.1 Pea Pod Flour Fermentation

Fermentation is a well‐studied process with notable effects on the nutritional composition and functional properties of various legumes. However, PPF remains a relatively underexplored by‐product. Therefore, the viability of both *Lactiplantibacillus plantarum* DD_T_B61 and *Lacticaseibacillus casei* DD_B_B71, as well as the pH, were assessed at 6 h, 24 h, and 48 h of fermentation of PPF.

**FIGURE 1:**
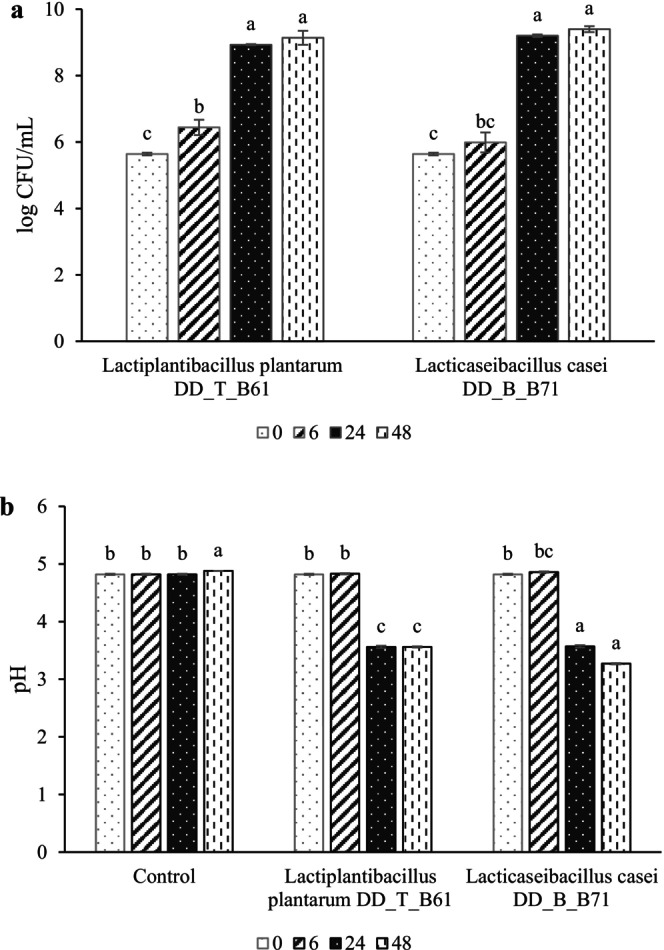
Variations in the cell viability (a) and pH (b) during the fermentation period. Different letters in the same partition are statistically different (*p* < 0.05).

The viable counts observed at 6 h indicated that PPF supported the survival of both isolates during the initial stage of fermentation, while LAB populations ranged from 5.64 ± 0.04 to 9.40 ± 0.09 log CFU/mL throughout the fermentation period (Figure 1a). Similarly, *Lactiplantibacillus plantarum* ITM21B increased from 5.00 ± 0.00 to 8.49 ± 0.33 log CFU/g after 14 h of sub‐standard fresh green peas fermentation (Di Biase et al. 2025), supporting the ability of pea‐derived substrates to sustain LAB growth during fermentation. While the highest populations of *Lactiplantibacillus plantarum* DD_T_B61 (9.14 ± 0.21 log CFU/mL) and *Lacticaseibacillus casei* DD_B_B71 (9.40 ± 0.09 log CFU/mL) were observed after 48 h of fermentation, no statistically significant difference in cell counts was detected between 24 and 48 h of either isolate (*p* > 0.05).

Throughout fermentation, the pH of PPF ranged from 3.27 to 4.86, with the lowest value recorded after 48 h of fermentation by *Lacticaseibacillus casei* DD_B_B71. However, no significant differences in pH were observed between 24 and 48 h of fermentation (*p* > 0.05) (Figure 1b). A similar decrease in pH has been reported during the fermentation of sub‐standard green peas with *Lactiplantibacillus plantarum* ITM21B, where pH decreased from 6.48 ± 0.27 to 4.43 ± 0.08 after 14 h (Di Biase et al. 2025), as well as during the fermentation of fava bean by‐products (Omar et al. 2021). Similarly, the stable bacterial populations during this period may be attributed to the accumulation of organic acids and the resulting acidic conditions, which may have restricted further bacterial growth while maintaining cell viability. This is consistent with Zhang et al. (2024) indicating that differences in acid tolerance among *Lactobacillus* strains influence bacterial growth during fermentation.

### 3.2 The Effect of Fermentation on Total Phenolic Content of Pea Pod Flour

The total phenolic content (TPC) of different legume by‐products has been investigated, including fava bean (Çalışkantürk Karataş et al. 2017), chickpea (Johnson et al. 2021; Niño‐Medina et al. 2017), soybean (Niño‐Medina et al. 2017), and cowpea (Traffano‐Schiffo et al. 2020); however, comparatively fewer studies have evaluated the effects of fermentation on TPC of legume by‐products. However, considering the studies conducted on legumes, including chickpea flour (Sáez et al. 2022), chickpea flour with seed coats (Sánchez‐Magaña et al. 2014), dehulled black soybean extracts (Cheng et al. 2013), the general trend indicates that fermentation increases TPC, although inconsistent findings reporting decreases have also been observed in certain cases.

In general, the TPC of the PPF used in the present study (11.07 ± 0.92 mg GAE/g) was within the range of values reported for pea pod‐derived materials in the literature (Table 1). A comparable TPC (8.22 mg GAE/g) was reported by Castaldo et al. (2022) for water‐based pea pod extracts, while Babbar et al. (2014) found values ranging from 8.0 ± 0.34 to 13.6 ± 0.20 mg GAE/g DW depending on the extraction solvent, highlighting the considerable influence of extraction conditions on phenolic extraction. Likewise, Hadrich et al. (2014) reported a broad TPC range (8.75–67.5 mg GAE/g) for pea peel extracts, further demonstrating that extraction methodology and sample preparation may have a great impact on phenolic content. Other studies have also demonstrated that pea pod‐derived materials are rich sources of phenolic compounds (Ben Said et al. 2025; Estivi et al. 2025; Mejri et al. 2019; Sik et al. 2024). However, variations in TPC among studies are expected, as the content and composition of phenolic compounds are influenced not only by extraction methodology but also by cultivar, growing conditions, environmental factors, and post‐harvest processing (Jovanović et al. 2024).

**TABLE 1:** Effect of fermentation on TPC and antioxidant activity on PPF.

Microorganism	Fermentation period (h)	Total phenolic content (mg GAE/g)	Antioxidant activity	
			DPPH (mg TE/g)	CUPRAC (mg TE/g)
PPF	0 (control)	11.07 ± 0.92^bc^	1.91 ± 0.35^e^	7.88 ± 0.62^ab^
	6	12.01 ± 0.75^ab^	2.63 ± 0.49^d^	8.01 ± 0.46^ab^
	24	11.57 ± 0.93^abc^	3.27 ± 0.33^c^	8.01 ± 0.72^ab^
	48	12.17 ± 0.72^ab^	4.33 ± 0.46^b^	6.95 ± 0.68^b^
F1‐PPF	6	10.35 ± 0.93^c^	4.16 ± 0.37^b^	7.13 ± 0.47^b^
	24	11.75 ± 0.67^ab^	2.70 ± 0.29^d^	7.62 ± 0.73^ab^
	48	10.97 ± 0.74^bc^	2.67 ± 0.39^d^	7.57 ± 0.83^ab^
F2‐PPF	6	12.23 ± 0.42^ab^	3.17 ± 0.39^cd^	6.85 ± 0.75^b^
	24	11.31 ± 0.36^bc^	5.44 ± 0.23^a^	8.53 ± 0.74^a^
	48	12.82 ± 1.42^a^	5.13 ± 0.38^a^	8.55 ± 0.78^a^

*Note:* Different letters in the same column are statistically different (*p* < 0.05).

Abbreviations: F1‐PPF, fermented pea pod flour with *Lactiplantibacillus plantarum* DD_T_B61; F2‐PPF, fermented pea pod flour with *Lacticaseibacillus casei* DD_B_B71; PPF, pea pod flour.

Following fermentation, the TPC values of PPF ranged from 10.35 ± 0.93 to 12.82 ± 1.42 mg GAE/g. Although an increasing trend was observed in some fermentation treatments, the effect of fermentation on TPC was dependent on both the microorganism and fermentation duration. Compared with the PPF (11.07 ± 0.92 mg GAE/g), fermentation with *Lacticaseibacillus casei* DD_B_B71 resulted in a significant increase in TPC after 48 h, reaching the highest value (12.82 ± 1.42 mg GAE/g). In contrast, fermentation with *Lactiplantibacillus plantarum* DD_T_B61 did not significantly enhance TPC (*p* > 0.05), with values remaining between 10.35 ± 0.93 and 11.75 ± 0.67 mg GAE/g throughout fermentation. These findings indicate that the ability of LAB to modify the phenolic profile of PPF may vary among isolates, likely due to differences in their enzymatic activities and capacity to release bound phenolic compounds (Hou et al. 2025). Similarly, previous studies have reported increases in TPC following fermentation of legume‐based substrates. Di Biase et al. (2025) demonstrated that fermentation of sub‐standard green peas with *Lactiplantibacillus plantarum* ITM21B for 14 h increased TPC from 13.92 ± 0.50 to 19.08 ± 0.38 mg GAE/L. However, certain phenolic compounds may be metabolized by different LAB strains, potentially resulting in a reduction in TPC (De Montijo‐Prieto et al. 2023), as observed during fermentation with *Lactiplantibacillus plantarum* DD_T_B61.

Although fermentation by LAB can promote the release of bound phenolic compounds from plant matrices, the extent of this effect depends on the balance between phenolic release and microbial transformation (Landete et al. 2021). Several phenolic compounds previously identified in pea pod and hull, including phenolic acids such as 5‐caffeoylquinic acid (59.87 mg/100 g), quinic acid (7.20 mg/g) (Castaldo et al. 2022), *p*‐coumaric acid (6.60 ± 0.41 mg/100 g) (Sik et al. 2024), as well as flavonoids such as catechin, epicatechin, naringenin, hesperidin, rutin, and quercetin (Castaldo et al. 2022; Guo et al. 2019; Mejri et al. 2019; Sik et al. 2024), may undergo microbial transformation depending on the LAB strain and fermentation conditions. Therefore, the limited changes observed in TPC after fermentation with *Lactiplantibacillus plantarum* DD_T_B61 and *Lacticaseibacillus casei* DD_B_B71 may be attributed to a balance between the release of phenolics and their utilization.

### 3.3 The Effect of Fermentation on Antioxidant Activity of Pea Pod Flour

Antioxidant activity is one of the most important functional properties of plant‐derived materials. Therefore, the effect of fermentation on the antioxidant capacity of PPF was evaluated using both DPPH and CUPRAC assays. The antioxidant activity of PPF was determined to be 1.91 ± 0.35 mg TE/g and 7.88 ± 0.62 mg TE/g by DPPH and CUPRAC assays, respectively (Table 1). Although studies on the antioxidant activity of PPF are limited, Ben Said et al. (2025) and González‐Montemayor et al. (2021) reported DPPH radical scavenging activity values of 267.96 ± 1.29 mg TE/100 g DW and 21.12 ± 6.06 mg TE/g, respectively. The variations in antioxidant activity may be explained by the same factors contributing to the differences in TPC.

The antioxidant activities determined by the CUPRAC assay were consistently higher than those obtained by the DPPH assay for all samples, which is likely attributable to the different reaction mechanisms of the two methods (Capanoglu et al. 2008). While the DPPH assay evaluates the free radical‐scavenging capacity of compounds capable of donating hydrogen atoms or electrons, the CUPRAC assay measures the reducing capacity of antioxidants through the reduction of Cu(II)‐neocuproine complexes (Capanoglu et al. 2018). Despite these methodological differences, the highest antioxidant activities in both assays were observed after 24–48 h of fermentation with *Lacticaseibacillus casei* DD_B_B71, with no significant difference between these fermentation periods (*p* > 0.05).

PP have been reported to contain phenolic compounds such as catechin, epicatechin, and quercetin, which exhibit strong radical‐scavenging activity (Baranwal et al. 2022; Qi et al. 2022). As *Lactiplantibacillus plantarum* has been reported to metabolize certain phenolic compounds, including flavonoids with high radical‐scavenging activity (Pulido‐Mateos et al. 2024), the comparatively lower DPPH activity observed during fermentation with *Lactiplantibacillus plantarum* DD_T_B61 may be associated with the transformation of these highly reactive compounds.

Based on these findings, *Lacticaseibacillus casei* DD_B_B71 was selected for the production of F‐PPF used in the further analysis.

### 3.4 Color Values and Organic Acid Content of Pea Pod Flour

The color parameters (L*, a*, b*) and total color difference (ΔE) of PPF and F‐PPF are presented in Table 2. F‐PPF exhibited a lower ΔE than PPF, suggesting that fermentation contributed to better color preservation. However, both PPF and F‐PPF showed lower L* values than WF, reflecting the inherently darker color of PP. These findings are consistent with those reported by Nasir et al. (2023), who determined L*, a*, and b* values of 60.26 ± 0.84, −4.25 ± 0.15, and 23.01 ± 0.15, respectively, for PP powder.

**TABLE 2:** Color values and organic acid contents of flours.

		WF	PPF	F‐PPF
Color values	L*	91.86 ± 0.54^a^	63.59 ± 0.02^c^	69.62 ± 0.10^b^
	a*	−0.49 ± 0.04^a^	−3.54 ± 0.03^b^	−4.16 ± 0.03^c^
	b*	10.93 ± 0.41^c^	32.40 ± 0.03^a^	30.22 ± 0.32^b^
	ΔE	—	35.63	29.67
Organic acid content (mg/g)	Tartaric acid	0.54 ± 0.01^b^	17.58 ± 1.95^a^	16.26 ± 0.35^a^
	Lactic acid	ND	ND	59.22 ± 0.30
	Acetic acid	ND	34.78 ± 0.65^b^	136.93 ± 0.10^a^
	Citric acid	ND	0.54 ± 0.27^b^	13.28 ± 0.02^a^
	Oxalic acid	2.30 ± 0.00^b^	3.63 ± 0.26^a^	3.71 ± 0.00^a^

*Note:* Different letters in the same row are statistically different (*p* < 0.05).

Abbreviations: F‐PPF, fermented pea pod flour; ND, not detected; PPF, pea pod flour; WF, wheat flour; ΔE, color difference.

Although several studies have reported a decrease in L* value accompanied by an increase in the browning index following LAB fermentation (Adebo 2026; Du et al. 2023; Sobowale et al. 2024), findings consistent with the present study, demonstrating an increase in L* value after fermentation, have also been reported (Olvera‐Ortiz et al. 2025). This result may be attributed to the microbial metabolism of sugars and other compounds involved in oxidative reactions during fermentation. Furthermore, Olvera‐Ortiz et al. (2025) reported that an increase in the L* value may be advantageous for bakery products.

Organic acids are among the major metabolites produced during lactic acid fermentation and play a crucial role in determining the physicochemical properties, microbial stability, and sensory characteristics of fermented foods (Islam et al. 2024). Therefore, the changes in organic acid composition of PPF during fermentation were investigated (Table 2). In the present study, ascorbic acid, malic acid, butyric acid, and succinic acid were not detected in either PPF or F‐PPF. Along with this, while lactic acid content had reached 59.22 ± 0.30 mg/g after fermentation, the acetic acid content of F‐PPF was ~4‐fold higher than that in PPF. Unlike WF, both PPF and F‐PPF contain acetic and citric acids at different concentrations.

The relatively high concentrations of citric, tartaric, and oxalic acids observed in PPF may be attributed to the fact that these carboxylic acids are among the predominant organic acids naturally present in vegetable by‐products (Nasir et al. 2023). Consistent with the known metabolic characteristics of *Lacticaseibacillus casei*, which is capable of producing acetic acid under certain fermentation conditions (Wu et al. 2024), the acetic acid content increased markedly from approximately 34.78 ± 0.65 to 136.93 ± 0.10 mg/g following fermentation. While acetic acid is an endogenous metabolite in plants that serves as a metabolic intermediate and signaling molecule, particularly under abiotic stress conditions (Kudo et al. 2023; Rahman et al. 2024), its concentration generally increases during microbial fermentation (Rajendran et al. 2023). Similar increases in fermentation‐derived organic acids have also been reported in pea by‐products. Di Biase et al. (2025) reported the production of lactic acid (29.09 ± 0.56 mmol/kg) and acetic acid (4.41 ± 0.63 mmol/kg) after 14 h fermentation by *Lactiplantibacillus plantarum* ITM21B. Likewise, Estivi et al. (2025) reported an acetic acid concentration of 20.4 mg/g DM in pea by‐product fermented with *Propionibacterium freudenreichii* DSM 20271 for 72 h.

### 3.5 Cookie Characterization

WF is the key ingredient in cookie production because of its desirable techno‐functional characteristics. However, its gluten content restricts its consumption by individuals with gluten‐related disorders (Fernandes Almeida et al. 2025). In this context, PP represents a promising alternative, not only for the valorisation of agro‐industrial by‐products but also for the production of gluten‐free bakery products. Since fermentation may modify the different properties of PPF, cookies prepared with PPF fermented with *Lacticaseibacillus casei* DD_B_B71 were evaluated as an alternative to WF in gluten‐free cookie production. The physicochemical and color characteristics of all cookies are presented in Table 3.

**TABLE 3:** Physicochemical and color characteristics of cookies.

		WF‐C	PPF‐C	F‐PPF‐C
Texture analysis	Hardness (g)	3360.83 ± 47.76^a^	821.67 ± 36.98^b^	390.31 ± 36.44^c^
Physico‐chemical properties	Thickness (mm)	11.46 ± 0.52^a^	3.00 ± 0.22^c^	9.50 ± 0.70^b^
	Weight (g)	5.57 ± 0.39^a^	4.06 ± 0.14^c^	4.73 ± 0.45^b^
	Spread ratio (*D*/*T*)	2.84 ± 0.15^c^	10.42 ± 0.62^a^	3.53 ± 0.14^b^
	Baking loss (%)	17.24 ± 3.92^c^	37.66 ± 1.53^a^	27.46 ± 3.80^b^
	Water activity (a_w_)	0.34 ± 0.02^c^	0.46 ± 0.01^b^	0.50 ± 0.02^a^
	Moisture (%)	2.07 ± 0.14^a^	0.97 ± 0.01^b^	1.92 ± 0.03^a^
	pH	6.27 ± 0.15^a^	5.05 ± 0.01^b^	3.95 ± 0.09^c^
Color values	L*	79.64 ± 0.13^a^	42.52 ± 1.03^b^	39.94 ± 0.93^c^
	a*	−1.84 ± 0.02^b^	−0.83 ± 0.15^a^	−1.06 ± 0.24^a^
	b*	24.28 ± 0.02^c^	31.08 ± 0.54^b^	32.77 ± 0.68^a^
	ΔE	—	37.75	40.61

*Note:* Different letters in the same row are statistically different (*p* < 0.05).

Abbreviations: F‐PPF‐C, fermented pea pod flour cookie; PPF‐C, pea pod flour cookie; WF‐C, wheat flour cookie.

The incorporation of PPF significantly reduced cookie hardness compared with WF (*p* < 0.05), and the hardness decreased from 3360.83 ± 47.76 g in WF‐C to 821.67 ± 36.98 g and 390.31 ± 36.44 g in PPF‐C and F‐PPF‐C, respectively. Compared with products prepared using WF, the partial or complete replacement of WF with alternative flours has generally been reported to reduce the hardness of bakery products, including bread (Belghith Fendri et al. 2016) and cookies (Ahmad et al. 2016). The reduced hardness may be attributed to the absence of gluten in PPF (Giri and Sakhale 2019), while fermentation could have further softened the flour matrix through microbial modification of polysaccharides and other structural components (Qi et al. 2020) resulting in a more fragile cookie structure.

The incorporation of PPF also altered the physicochemical properties of cookies. Compared with WF‐C, PPF cookies exhibited lower thickness and higher spread ratio, indicating reduced dough resistance during baking. Fermentation affected thickness while decreasing the spread ratio, suggesting that fermentation modified the water‐binding behavior and structural characteristics of PPF. Similarly, baking loss was significantly higher in cookies containing PPF but decreased following fermentation, indicating improved moisture retention during baking. These results are consistent with previous studies demonstrating that the incorporation of legume‐ and vegetable‐derived flours influences the dimensional characteristics of cookies. Arise et al. (2025) reported a decrease in cookie thickness after the incorporation of chickpea flour, while Kaur et al. (2023) similarly observed reduced thickness in cookies containing pea peel powder.

Despite exhibiting lower baking loss than PPF‐C, F‐PPF‐C showed the highest water activity and moisture content. This finding suggests that fermentation enhanced the water‐holding capacity of flour, most likely through modifications in dietary fiber and other hydrophilic constituents during microbial fermentation (Badia‐Olmos et al. 2024; Ojha et al. 2018).

As expected, fermentation significantly reduced the pH of cookies because of the accumulation of organic acids, particularly lactic and acetic acids. A similar trend was reported by Sáez et al. (2022), who reported a lower pH in crackers produced with fermented chickpea flour (5.6 ± 0.1) compared with crackers prepared from unfermented flour (7.3).

Color is one of the most important quality attributes influencing consumer preferences for bakery products. Therefore, the effect of PPF incorporation and fermentation on cookie color was evaluated by determining the L*, a*, b*, and total color difference (ΔE) values (Table 3). Cookies prepared with PPF exhibited markedly lower L* values than WF‐C, reflecting the naturally darker color of PPF. These changes are likely associated with modifications in pigment composition together with enhanced Maillard reactions promoted by the presence of fermentation‐derived metabolites during baking. In previous studies, the darker color observed in breads enriched with pea and broad bean pod fibers (Belghith Fendri et al. 2016) and in cookies containing pea peel powder (Kaur et al. 2023) was attributed to the formation of Maillard reaction products during baking. Similarly, significantly lower L* values than the control were reported for both the crumb and crust of muffins enriched with PP powder (Pooja et al. 2022) and for breads containing fermented pea by‐products (Estivi et al. 2025). Although the general trend was towards darker colors in cookies, muffins, and breads enriched with pea or its by‐products after baking, no statistically significant difference in lightness was observed between bread enriched with PPF and the control (González‐Montemayor et al. 2021). In contrast to the decrease in L* value, the incorporation of F‐PPF increased both the a* (−1.06 ± 0.24) and b* (32.77 ± 0.68) values of cookies in the present study. Similar findings were reported by Estivi et al. (2025) for breads enriched with fermented pea by‐products. Specifically, the incorporation of fermented pea by‐products resulted in higher b* values in the bread crumb, indicating a more pronounced yellow hue, whereas the bread crust exhibited lower b* values than the white bread control.

### 3.6 Total Phenolic Content and Antioxidant Activity of Cookies

The effect of F‐PPF on the TPC of cookies was also evaluated. Cookies prepared with WF exhibited the highest TPC (6.55 ± 0.31 mg GAE/g), whereas the TPC of cookies produced with PPF and F‐PPF was approximately 17%–18% lower (Table 4). Notably, the TPC of both PPF and F‐PPF (~11–12 mg GAE/g) was considerably higher than that of the corresponding cookies (~5–5.5 mg GAE/g). Besides the thermal degradation and oxidation during baking, the lower TPC of cookies compared with the corresponding flours may also be attributed to other ingredients in the final baked product.

**TABLE 4:** Total phenolic content and antioxidant activity of cookies.

Samples	Total phenolic content (mg GAE/g)	Antioxidant activity (mg TE/g)	
		DPPH	CUPRAC
WF‐C	6.55 ± 0.31^a^	0.51 ± 0.25^b^	0.42 ± 0.11^c^
PPF‐C	5.34 ± 0.21^b^	1.57 ± 0.19^a^	4.04 ± 0.49^a^
F‐PPF‐C	5.45 ± 0.25^b^	1.46 ± 0.23^a^	2.75 ± 0.37^b^

*Note:* Different letters in the same column are statistically different (*p* < 0.05).

Abbreviations: F‐PPF‐C, fermented pea pod flour cookie; PPF‐C, pea pod flour cookie; WF‐C, wheat flour cookie.

To the best of our knowledge, only a limited number of studies have investigated the incorporation of pea by‐products into bakery products. Most of these studies have evaluated the partial substitution of WF with pea by‐products in bread formulations. Sik et al. (2024) reported TPC values of 126 ± 3.74 and 153 ± 6.17 mg GAE/100 g in flat breads enriched with PP powder at different substitution levels, although these values were considerably lower than those obtained in the present study. Furthermore, no significant differences in TPC were observed between the control wheat bread and breads containing whole PP powder. Similarly, Estivi et al. (2025) reported TPC values of 197.28 ± 1.99 and 214.54 ± 0.59 μg/g for breads enriched with 20% unfermented and fermented pea by‐products, respectively, with fermentation resulting in a slight increase in TPC. A comparable trend was observed in breads supplemented with a bioingredient obtained by fermenting sub‐standard green peas with *Lactiplantibacillus plantarum* ITM21B, which exhibited higher TPC values than the control bread (Di Biase et al. 2025).

The incorporation of PPF significantly increased the antioxidant activity of cookies, as demonstrated by both DPPH radical‐scavenging activity (1.46–1.57 mg TE/g) and CUPRAC reducing capacity (2.75–4.04 mg TE/g), compared with WF‐C (Table 4). In line with the present study, breads supplemented with a bioingredient obtained by fermenting sub‐standard green peas with *Lactiplantibacillus plantarum* ITM21B exhibited significantly higher DPPH radical‐scavenging activity (1.95 ± 0.02 μmol TE/g) than the control bread (1.24 ± 0.03 μmol TE/g).

### 3.7 Organic Acid Content of Cookies

The organic acid profile of cookies is exhibited in Table 5, and ascorbic acid, malic acid, butyric acid, and succinic acid were not specified in either type of cookie. Lactic acid was detected exclusively in cookies prepared with F‐PPF, whereas acetic acid was present in both PPF‐C and F‐PPF‐C, which reached significantly higher concentrations following fermentation. Citric acid was detected only in F‐PPF‐C, while tartaric and oxalic acids were identified in all cookie samples. The overall organic acid profile of cookies was consistent with that observed in the corresponding flours.

**TABLE 5:** Organic acid contents of cookies.

Organic acids (mg/g sample)	WF‐C	PPF‐C	F‐PPF‐C
Tartaric acid	0.15 ± 0.00^c^	6.31 ± 0.21^b^	10.49 ± 0.24^a^
Lactic acid	ND	ND	10.46 ± 1.13
Acetic acid	ND	39.29 ± 0.84^b^	70.95 ± 5.00^a^
Citric acid	ND	ND	4.13 ± 0.47
Oxalic acid	1.13 ± 0.00^c^	2.03 ± 0.06^a^	1.80 ± 0.02^b^

*Note:* Different letters in the same row are statistically different (*p* < 0.05).

Abbreviations: F‐PPF‐C, fermented pea pod flour cookie; ND, not detected; PPF‐C, pea pod flour cookie; WF‐C, wheat flour cookie.

The retention of fermentation‐derived organic acids in the final baked product may be advantageous, as organic acids are widely used in food systems to improve microbial stability and potentially extend shelf life (Su et al. 2019). However, excessive accumulation of certain organic acids, particularly acetic acid, may also influence the sensory properties of the product by imparting sour or vinegar‐like flavor (Li et al. 2021), thereby negatively affecting consumer acceptability. Although fermentation markedly increased the acetic acid content of PPF, its concentration decreased after baking, most likely because of the partial volatilization during thermal processing.

### 3.8 Sensorial Characteristics of Cookies

**FIGURE 2:**
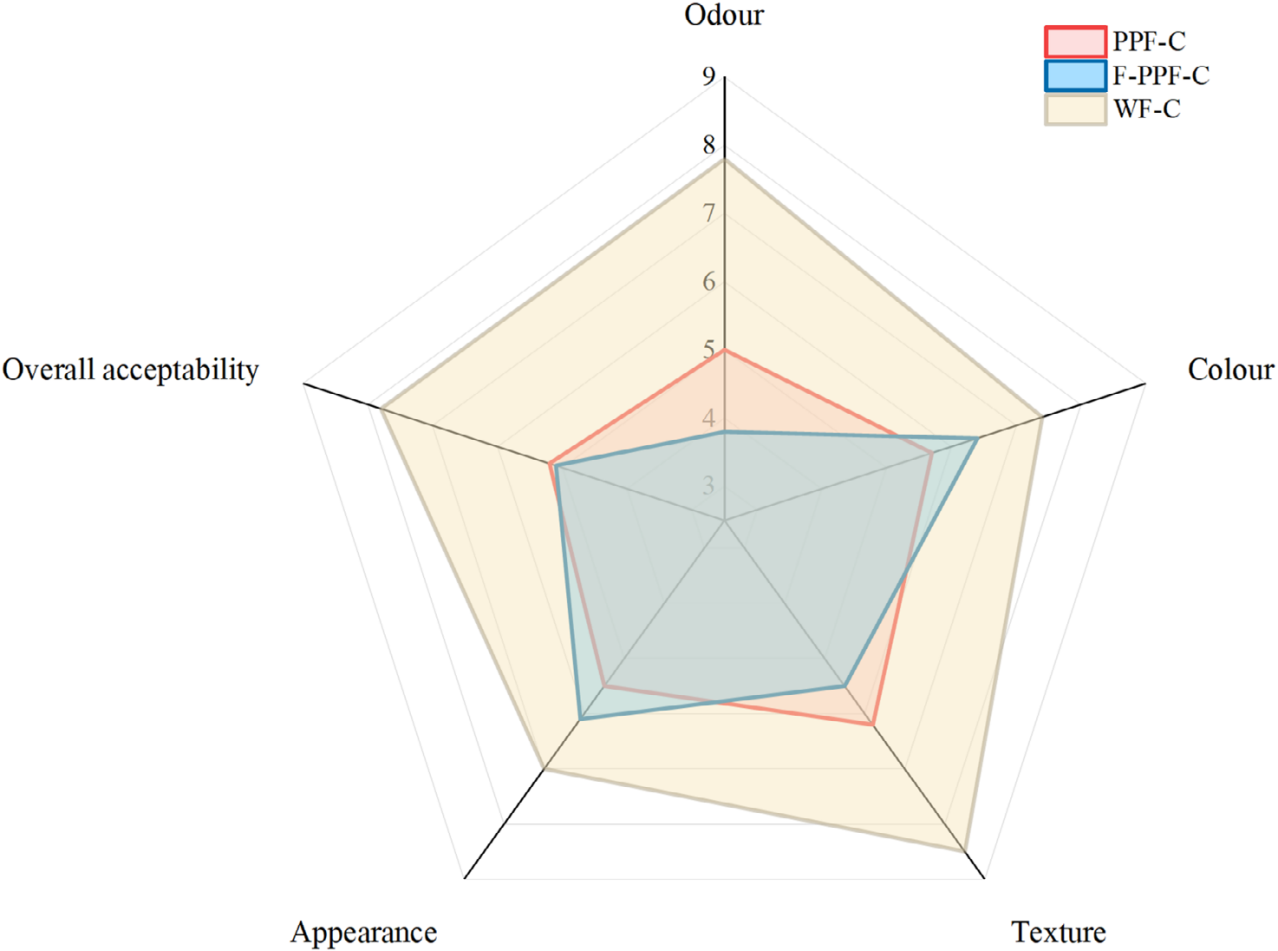
Radar diagram of cookies based on sensorial analysis. F‐PPF‐C, fermented pea pod flour cookie; PPF‐C, pea pod flour cookie; WF‐C, wheat flour cookie.

Sensory evaluation was performed to assess the consumer acceptability of cookies based on odor, color, texture, appearance, and overall impression (Figure 2). Among all prepared cookies, WF‐C received the highest scores for every sensory attribute, indicating greater overall acceptability. The lower acceptability of cookies PPF may primarily be attributed to their darker color, which has previously been identified as a major factor negatively influencing the sensory acceptance of pea pod‐enriched cookies (Kaur et al. 2023).

When the cookies prepared by both PPF and F‐PPF were compared, PPF‐C received higher scores for odor, texture, and overall acceptability, whereas F‐PPF‐C was preferred in terms of color and appearance. The lower odor score of F‐PPF‐C may be associated with the higher concentration of fermentation‐derived organic acids. Similar reductions in overall acceptability have also been reported for flatbreads enriched with PP powder (Sik et al. 2024). Nevertheless, the sensory acceptability of F‐PPF‐C could be further improved by optimizing the formulation and fermentation conditions. Adjustments in the fermentation process to control organic acid production, together with the incorporation of flavor‐masking ingredients and sweeteners may enhance the sensory properties while preserving the nutritional and functional benefits of F‐PPF. Furthermore, the present study was based on the use of 100% F‐PPF. Future research could explore the incorporation of different gluten‐free flours, either alone or in combination with F‐PPF, to develop novel gluten‐free products with improved sensory quality while maintaining their nutritional and functional advantages.

### 3.9 Correlation of Various Parameters of Cookies

**FIGURE 3:**
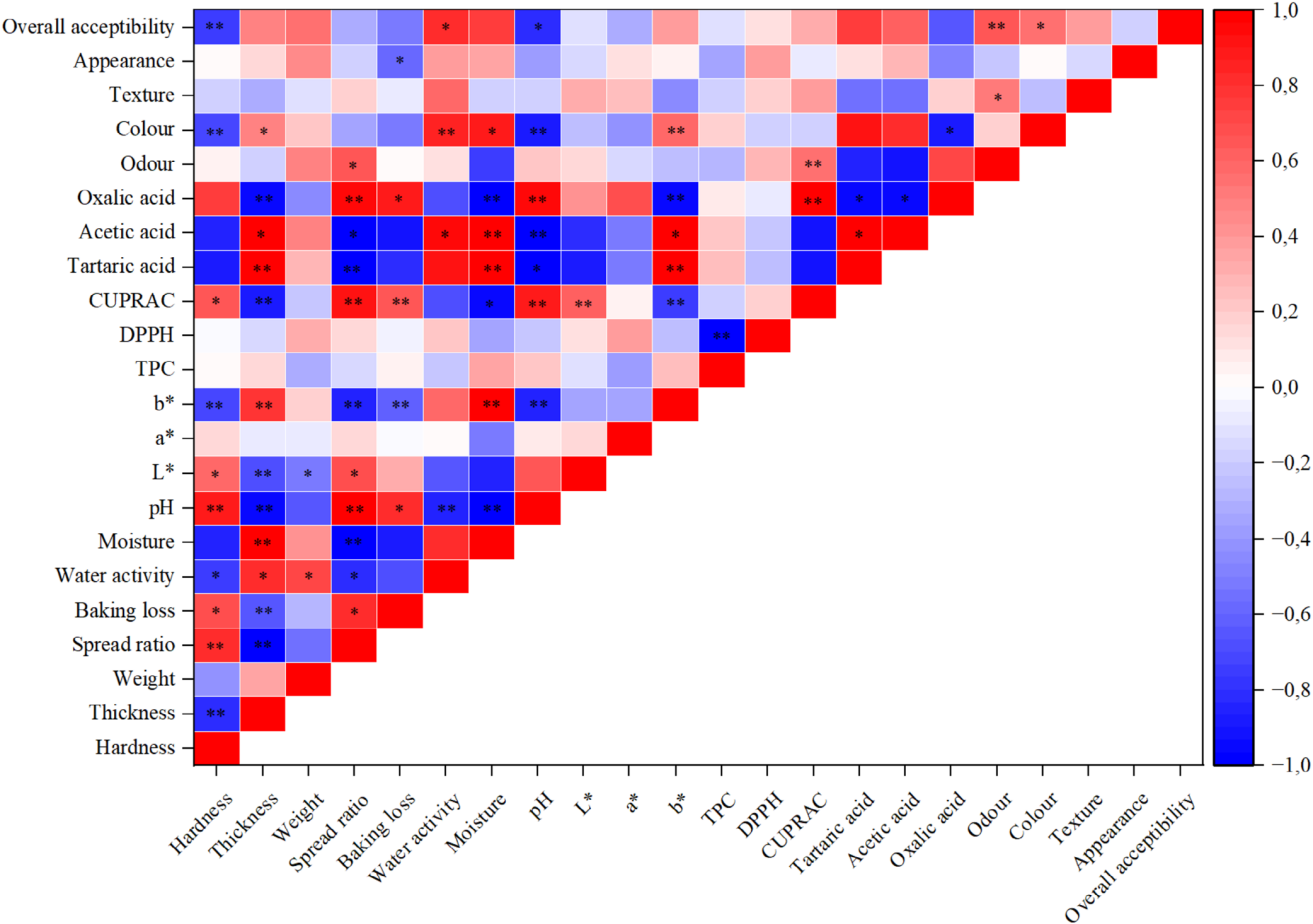
Pearson correlations between physicochemical, nutritional, color, antioxidant, organic acid, and sensory characteristics of PPF‐C and F‐PPF‐C (**Correlation is significant at the 0.01 level (2‐tailed); *Correlation is significant at the 0.05 level (2‐tailed)).

Pearson's correlation coefficient was used to assess the correlations among all tested parameters of cookies prepared with PPF and F‐PPF. As shown in Figure 3, strong correlations were observed among the physicochemical, nutritional, color, antioxidant, organic acid, and sensory characteristics of the cookies. Firstly, significant correlations were observed between hardness, water activity, and pH and the overall impression of cookies. However, the correlations were positive for water activity (*p* < 0.05) and negative for hardness (*p* < 0.01) and pH (*p* < 0.05). Despite the positive correlation between hardness and overall acceptability previously reported for biscuits (Boğa et al. 2025), the present study revealed a negative relationship between hardness and overall acceptability (r = −0.766). Consistent with the current findings, negative and positive relationships between spread ratio and hardness, and thickness and hardness, respectively, have previously been reported for cookies containing guava flour (Mahajan et al. 2024) and wheat bran (Kong et al. 2022). Moisture was correlated with several physical and color parameters, antioxidant activity, and organic acids, underscoring its importance in cookie‐type bakery products. TPC showed no significant correlation with the other parameters, except for DPPH, with which it exhibited a strong negative correlation (*r* = −1, *p* < 0.01). Although oxalic acid showed negative correlations with thickness, moisture, and b* value (*p* < 0.01), a positive correlation was observed between spread ratio, pH, and CUPRAC (*p* < 0.01). Similarly, the lack of correlation between a* value and the other variables has also been reported for gluten‐free cookies. At the same time baking loss was not significantly correlated with the color values, except for the b* value (Zhang et al. 2025).

## 4 Conclusion

The incorporation of naturally gluten‐free legumes and their by‐products into product formulations represents an important strategy for developing sustainable gluten‐free products. In this context, the valorisation of PP by‐products offers an opportunity to produce value‐added ingredients with both economic and environmental benefits. However, the direct usage of PPF in bakery products is limited by its techno‐functional properties, highlighting the need for appropriate processing approaches. The present study demonstrated that the incorporation of both PPF and F‐PPF into cookies significantly enhanced their antioxidant capacity compared with WF‐C. Nevertheless, the sensory characteristics of PPF‐C and F‐PPF‐C remained lower than WF‐C, indicating that consumer acceptability remains a challenge. Overall, this research contributes to the development of cookies enriched with PPF, exhibiting enhanced functional and nutritional properties. The utilization of PP further enhances the relevance and sustainability of this approach. However, further studies are needed to investigate additional aspects, particularly the optimization of PPF substitution levels to overcome the limitations associated with its direct use.

## Author Contributions

**Funda Karbancioglu‐Guler:** investigation, conceptualization, resources, writing – review and editing. **Neris Danç:** methodology. **Dilara Devecioglu:** methodology, investigation, conceptualization, writing – original draft. **Berfin Kaya:** methodology. **Aysegul Mutlu‐Ingok:** investigation, writing – original draft.

## Funding

This work was supported by Türkiye Bilimsel ve Teknolojik Araştırma Kurumu, 1919B012320912 and the Scientific Research Council of Istanbul Technical University under grant number MDK‐2021‐43273.

## Conflicts of Interest

The authors declare no conflicts of interest.

## Data Availability

The authors confirm that the data supporting the findings of this study are available within the article. Raw data that support the findings of this study are available from the corresponding author upon reasonable request.
